# FDTD Simulations for Rhodium and Platinum Nanoparticles for UV Plasmonics

**DOI:** 10.3390/nano13050897

**Published:** 2023-02-27

**Authors:** Andrey Yurevich Zyubin, Igor Igorevich Kon, Darya Alexeevna Poltorabatko, Ilia Gennadievich Samusev

**Affiliations:** REC «Fundamental and Applied Photonics. Nanophotonics», Immanuel Kant Baltic Federal University, A. Nevskogo 14, 236016 Kaliningrad, Russia

**Keywords:** FDTD, SERS, simulations, nanoparticles, ultraviolet, optical sensor, surfaces

## Abstract

The article describes the results of finite-difference time-domain (FDTD) mathematical modeling of electromagnetic fields distortion near the surfaces of two transition metals: rhodium (Rh) and platinum (Pt) on glass (SiO_2_) substrates. Results were compared with calculated optical properties of classical SERS generating metals (Au and Ag). We have performed FDTD-based theoretical calculations for UV SERS-active nanoparticles (NPs) and structures based on hemispheres of Rh and Pt and planar surfaces, consisting of single NPs with varied gaps between them. The results have been compared with gold stars, silver spheres and hexagons. The prospects of the theoretical approach for single NPs and planar surfaces modeling to evaluate optimal field amplification and light scattering parameters have been shown. The presented approach could be applied as a basis for performing the methods of controlled synthesis for LPSR tunable colloidal and planar metal-based biocompatible optical sensors for UV and deep-UV plasmonics. The difference between UV-plasmonic NPs and plasmonics in a visible range has been evaluated.

## 1. Introduction

Over the last few decades, the development of optical sensors based on enhanced Raman scattering (RS) and appearing near noble metal surfaces has been a promising area of research [1]. Multiple amplification of the Raman scattering of light leads to the surface-enhanced Raman scattering (SERS) effect [2], which is widely used for weakly scattered signals from biological object detection [3]. One of the tasks of modern plasmonics and optosensorics is the creation of structures with certain optical properties for the study of specific objects, such as cells [4,5], proteins [6] and inorganic matter [7,8], including environmentally friendly materials [8]. Noble metals are widely used to manufacture such sensors due to the possibility of plasmon resonance generation near their surface under the influence of monochromatic radiation. Metals with the ability to generate plasmon resonance in the ultraviolet (UV) and infrared (IR) spectral ranges are believed to be promising [9,10]. It was revealed that the SERS signal enhancement and plasmon maximum position depends on the shape and size of NPs, the distance between them, as well as on their natural properties, material and other parameters.

Mathematical modeling methods are currently widely used for preliminary calculations of the optical properties of colloidal and planar nanostructures [11]. This step is important for the improvement and simplification of complex experimental synthesis processes and the more accurate prediction of the results. It is also important to calculate the optical properties of the structure when inducing the effect of localized surface plasmon resonance (LSPR) [12,13] in the “hot spot” zones. Nowadays, the development of biosensors is one of the promising tasks in sensorics, the effect of surface plasmon resonance has found its wide application here. Optical biosensors are used in the improvement of the diagnostics of diseases in medical institutions [14,15]. Moreover, researchers perform metal nanostructure applications for the implementation of metal enhanced fluorescence (MEF) [16,17,18,19]. In addition, due to the transfer of plasmonic energy, fluorescence amplification becomes possible, which makes it possible to detect low-fluorescent compounds and determine the in depth parameters of the fluorescence lifetime and quantum yield [20,21,22,23]. The advantages of MEF also include increased detectability and photostability of fluorophores. Investigations include spectral and time-resolved fields [24,25] and fluorescence quenching [26,27]. MEF has found its application in the detection of single molecules [28]. SERS is also implemented on various rough surfaces, in particular, on transition metals [29]; SERS on these metals has been successfully used to study surface adsorption, electrocatalysis and corrosion of various substrates [30]. Plasmonics in the UV wavelength range is considered to be a promising area of research for the study of the aromatic components of cells, proteins and tissues, since the laser excitation source wavelengths used for UV and deep-UV SERS lie outside the fluorescence excitation region of such samples. The usage of both Pt and Rh NPs opens up significant prospects for UV and deep-UV SERS. It is also worth noting that the electromagnetic enhancement is rather small in the UV region, where attenuation is usually high due to interband transitions. For example, despite metals showing the traditionally high electric field amplification in the visible and near IR ranges, they do not produce a sufficient SERS effect with laser excitation in the UV range. Although the optical properties of the transition metal differ from those of Ag and Au, they can be compared numerically in terms of the electric field *E* and the SERS enhancement factor (EF) value when using classical glass substrates as a substrate.

The choice of the geometric structure of the NPs deposited on the surface plays an important role in the creation of hot spots with strong electromagnetic fields in the UV regime. UV-SERS substrates were fabricated using electrodeposition in an electrochemical oxidation-reduction cycle or magnetron sputtering in transition metal systems (Ni, Ru, Rh, and Co), however, sometimes, such substrates exhibit low activity and weak signal uniformity [31,32,33,34]. However, in the UV region, based on applications, there are still no economical SERS substrates, which are characterized by simple preparation methods.

Aluminum (Al) can be a candidate for cheap and reliable sensors in the UV, but for biophysics research, it has two major drawbacks: toxicity [35,36,37] and oxidizability. Al NPs are very reactive to atmospheric oxygen, and a thin passivation layer of aluminum oxide (4 nm thickness or more) forms on any exposed aluminum surface in a matter of hundreds of picoseconds. It provides plasmon energy loss in NPs and creates a weak LSPR peak, as a result [38]. In this regard, studies in the general case of NPs and, in particular, substrate-surfaces in the UV range, both experimentally and by modeling methods, are not numerous [10,29].

Si NPs can also be used in the UV region and calculated with FDTD. As they have strong interband transitions, they lead to the negative permittivity of Si across the ultraviolet spectral range. Localized plasmon resonance can be excited in Si in the UV (at ∼250 nm wavelength) [33]. In [34], it was demonstrated that the generation of extreme ultraviolet plasmons on Si was not possible using other plasmonic materials, such as aluminum, silver, or gold. The authors proposed a simple Si/SiO_2_ multilayer stack with a hyperbolic isofrequency response that could generate tunable and broadband Cherenkov radiation in UV.

In our study, we have used two transition metals, Rh and Pt, on glass substrates and compared it with the calculated optical properties of substrates with NPs containing classical SERS generating metals (Au and Ag). We have performed FDTD-based theoretical calculations on UV SERS-active NPs and structures based on hemispheres of Rh and Pt, and compared the optical parameters with Au stars, silver spheres and hexagons. The difference in the optical properties in the UV range (for Rh, Pt) and Vis range (for Ag, Au) has been shown.

## 2. Computational Algorithm

Modeling and theoretical calculations were carried out using the Ansys Lumerical FDTD software and theoretical model, that were described in detail in our previous paper [10]. The calculation process was carried out in two stages. At the beginning, the optical properties for individual NPs of certain sizes were simulated in accordance with the experimental parameters when obtaining NPs in a colloidal solution. Further, these NPs were simulated on SiO_2_ surfaces. Spherical form of NP were replaced on hemisphere form. In this case, we created idealized rough surfaces with varied gaps between NPs, i.e., simulated various “hot-spot” zones (the places where the SERS effect occurs). On all surfaces, the distance between NPs was taken as 1, 2 and 3 nm. Simulation options were taken as follows:

(A) Pt spheres and surface consist of hemispheres with radii: 15, 20, 40 and 50 nm. Excitation by the monochromatic radiation source with λ = 244 and 355 nm was maintained;

(B) Rh spheres and surface consist of hemispheres with radii: 35, 40, 50 and 70 nm. Excitation by the monochromatic radiation source with λ = 244 nm and 355 nm was maintained;

(C) Ag spheres and surface consist of hemispheres with radii: 20, 30, 40 and 70 nm. Excitation by the monochromatic radiation source with λ = 532 and 632.8 nm was maintained;

(D) Hexagonal Ag structures and surface consist of hemispheres with radii: 20, 40, 50 and 70 nm. Excitation by the monochromatic radiation source with λ = 532 and 632.8 nm was maintained;

(E) Five-pointed Au stars and surfaces consisting of such NPs: (1) with an outer radius of 30 nm, an inner radius of 10 nm, and a height of 8 nm; (2) with an outer radius of 40 nm, an inner radius of 10 nm, a height of 8 nm; (3) with an outer radius of 45 nm, an inner radius of 15 nm, a height of 8 nm; (4) with an outer radius of 50 nm, an inner radius of 20 nm, a height of 10 nm; (5) with an outer radius of 60 nm, an inner radius of 20 nm, a height of 10 nm; (6) with an outer radius of 65 nm, an inner radius of 25 nm, a height of 10 nm; (7) with an outer radius of 75 nm, an inner radius of 25 nm, a height of 10 nm. Excited by the monochromatic radiation of 632.8 nm and 785 nm. The simulation process was performed as follows:

(1) The counting area, grid resolution and boundary conditions were set. For the computational domain, a rectangular grid was used from the basic Yee algorithm in the Cartesian coordinate system. The main modeling quantities (material properties, object geometries, electric and magnetic fields) were calculated separately at each grid point. The size of the computational region along the axes was in the same position, so that the dependence did not change during the propagation of the excitation. To maintain accuracy, the meshing algorithm generated a smaller mesh with a high index (to maintain a constant number of mesh points per wavelength). The minimum grid step was set to 0.25 nm. Then, an additional refinement grid was installed for modeling. The size of the computational region of the additional grid was set by the grid step: dx, dy, dz = 2.5 nm. We chose standard absorbing PML boundary conditions designed to absorb incident light with minimal reflections. Their parameters were as follows:

- Number of layers (for PML domain discretization purposes): 8;
- KAPPA, SIGMA, ALPHA (absorptive properties of PML regions kappa, sigma and alpha are estimated within the PML regions using polynomial functions) kappa = 2, sigma = 1, alpha = 0;
- Polinom (defines the order of the polynomial used to evaluate kappa and sigma): 3;
- Alpha-polynomial (defines the order of the polynomial used to evaluate the alpha channel): 1;
- Minimum and maximum number of layers (these provide an acceptable range of values for the number of PML layers). Minimum number of layers = 8, maximum number of layers = 64.

Physical parameters of simulation: travel time of a plane-polarized wave through the working zone was 1 ps, temperature was 300° K.

(2) A body with specified optical and geometric parameters was placed inside the counting region. Next, the optical and geometric parameters of the samples were set. We used materials from the Lumerical digital database for objects: (Ag, Au, Pt, Rh and SiO_2_) and changed their dimensional parameters for modeling. The values of such a parameter as the real and imaginary parts of the permittivity, which depends on the radiation frequency, were taken into account (Table 1). For Rh NPs: λ = 244 nm, Re (ε) = −4.64, Im (ε) = 3.62, λ = 355 nm, Re (ε) = −12.75, Im (ε) = 7.78.

**Figure 1:**
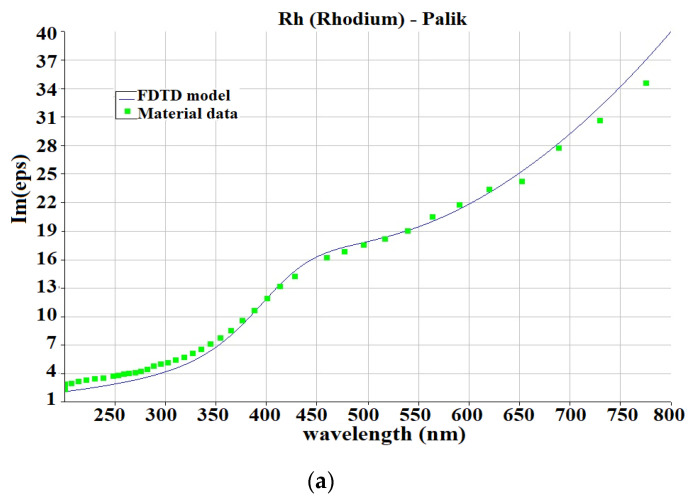

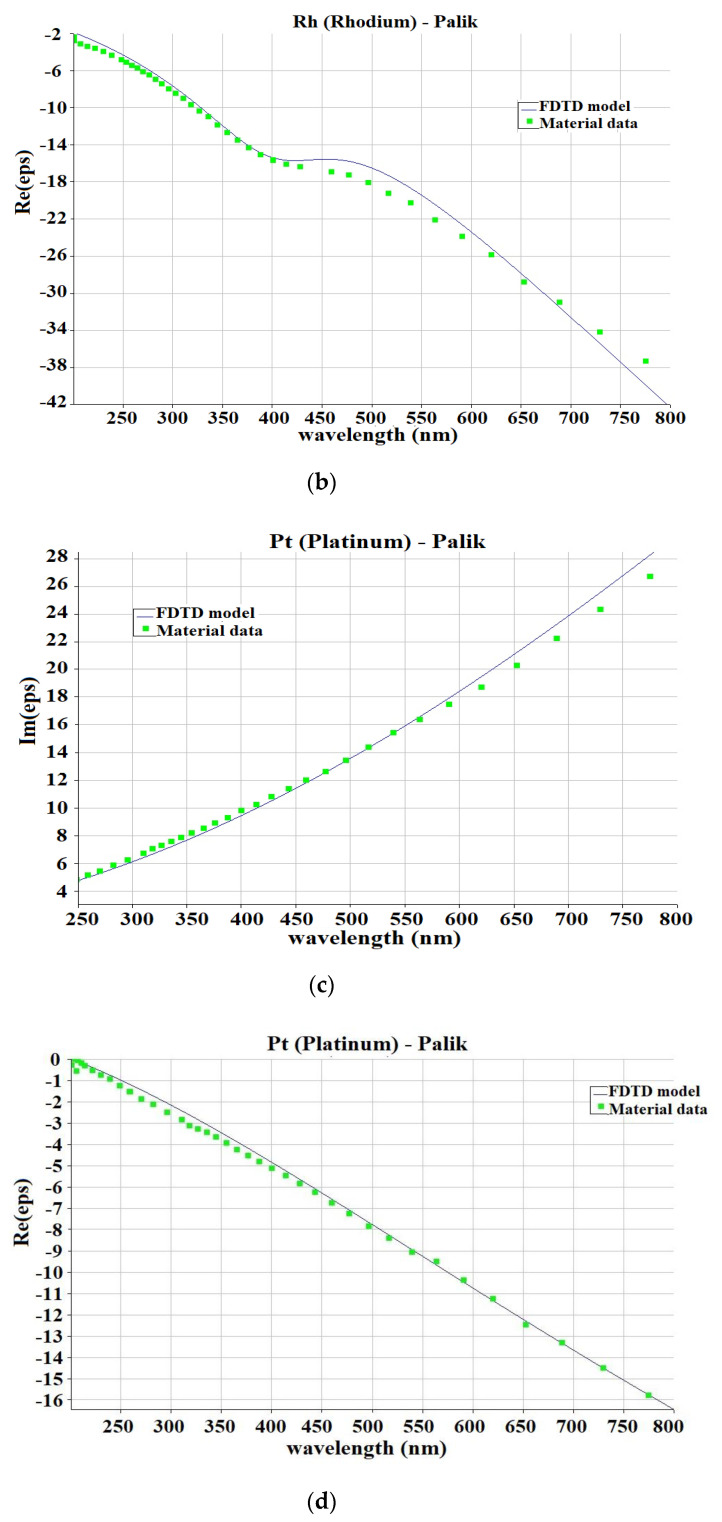
Re (ε) and Im (ε) graphs of permittivity for rhodium (**a**,**b**) and platinum (**c**,**d**).

The real and imaginary parts of the permittivity of rhodium are displayed in Figure 1a,b, and the real and imaginary parts of the permittivity of platinum are displayed in Figure 1c,d, including the values from the Lumerical database and FDTD line fit.

(3) At the next step, the parameters of the radiation source were set, primarily the wavelength. In our study, a total scattered field source (TFSF) was used, which is often suitable for studying the scattering of small NPs illuminated by a plane wave. The TFSF source divided the computational domain into two separate domains: (a) the total field domain, which included the sum of the incident field wave plus the scattered field, and (b) the scattered field domain, which included only the scattered field. The TFSF was an extended source. It is important to note that the physical field is a total field, and the split incident and scattered fields require careful interpretation. For NPs in a homogeneous medium, the incident field was a *p*—plane wave polarized wave. We obtained the magnitude of the electric tension in the maximum values.

(4) To provide the final information, the monitor plane was set parallel to the XZ plane, which gave the final information about the value of electric field *E* as a function of position in space, in the form of a 2D slice. The use of field monitors in the frequency domain allowed one to collect a field profile in this region and provide simulation results in some spatial domain to the FDTD solver. The direction of the p—polarized excitation wave was carried out by a plane wave polarized along the y axis, i.e., its direction corresponded to the normal vector to the monitor surface on the XZ axis (the image of the simulation along the XZ axes is shown below in the examples).

(5) Furthermore, the calculated values of electric field strength *E* were converted using the program code (scripts) into the intensity values of the SERS and the theoretical effective gain |E/E_0_|^4^ for SERS calculated in the XZ plane. We have changed the parameters of the modeling area in the script used to calculate the SERS and the EF coefficient, based on the dimensions of the surfaces (over the entire surface area). To recalculate the values, we used TFSF sources and modeled the parameters in the structure domain to find the maximum intensity and EF values. By and large, the calculation of SERS values made sense only for rough surfaces, however, the theoretical enhancement factor |E/E_0_|^4^ was also calculated for single NPs, which could be used to better evaluate the absorption peaks. This parameter, in contrast to experimental measurements, can be calculated without the conditions in the presence of the analyte—but from the point of view of quantum mechanical theory. The script code is presented in Appendix A. The script explanations are marked with the “#” symbol and in italics.

(6) As the last step, the optical parameters of rhodium (Rh) and platinum (Pt) were calculated: scattering, absorption and extinction (as a total index of absorption and scattering). The radiation source TFSF was set at a range of λ = 100–800 nm. As a result, on the *y*-axis, the values of the linear cross section indicator (cross section) were determined as the amount of optical power.

## 3. Simulation Results and Discussion

### 3.1. Ag Single Sphere NPs and Surfaces Consisting of Ag Hemispheres

We have interpreted the LPPR effect in terms of such parameters as the magnitude of local electric field strength *E* and the intensity of the SERS signals with the corresponding enhancement factor (EF) |*E*|^4^. Despite the fact that in Raman spectroscopy, the scattering intensity can be put into a linear dependence on the intensity of the incident field, as was written above *E*_0_^2^, and the square of the field strength |*E*_out|^2^ is outside the NP. Field strength *E* can, for example, be brought into line with the extinction spectrum. A complete understanding of the features of its characteristic spectra is not easy, due to the influence of the dielectric medium, size, as well as the width of the entire shape of the object and the half-height in the spectral position.

The SERS effect itself has a different, more complex nature and depends on the incident field plus the absorbed field, dipole and quadrupole interactions, the absorption of photon energy by a molecule, and inelastic resonant scattering; therefore, it does not directly depend on the value of field strength *E*. Moreover, the SERS is calculated over the entire surface of a significant widespread field and can take into account the total effect of the decision maker. Whereas the electric field strength, in our case, is a simpler indicator of plasmon amplification—numerically calculated over the entire region, and calculated as a function of position at a point. In the field inside the TFSF source, it depends both on the incident and scattered field. Moreover, in our study, we calculated the maximum *E* values, without trying to calculate the entire integral sum over the region of excitation propagation. However, both values in their own way characterize the behavior of the LSPR near the NPs. The SERS indicator, in this case, more clearly characterizes the sensory properties of the surfaces and makes it easy to correlate it with the experimental data obtained on the Raman. The SERS and EF values themselves are equivalent in our theoretical modeling, i.e., one value of SERS corresponds to only one value of EF. Although in experimental studies, there are inconsistencies between the theoretical values of EF, SERS and the experimental values of EF, due to the lack of their strict definitions; therefore, it is possible that they could be avoided in calculations on single molecules [34].

**Figure 2:**
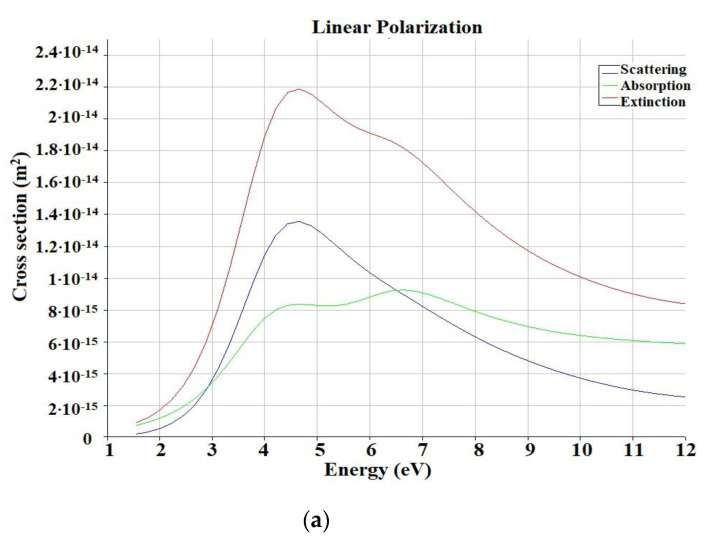

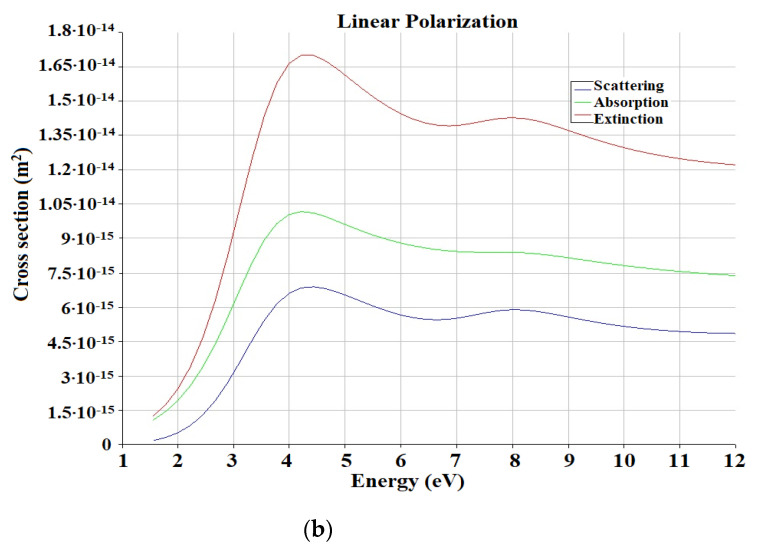
Scattering (blue line), absorption (green line) and extinction (red line) calculated cross section for the spherical Rh NPs (**a**) and Pt NPs (**b**).

In our paper, we also studied the LSPR effect that occurs on substrates in the gaps between NPs. Each simulation of NPs on substrates was accompanied by models of the same single NPs. As a result, we could evaluate the efficiency of the usage for certain NPs as substrates. The NPs were of various geometries: spherical, hexagonal and in the form of stars. Various metals were used, which were irradiated at a wavelength corresponding to the plasmon absorption peak of these metals. To determine the appropriate surface roughness coefficient, which would give the highest value of the LPR, we varied the distance between the NPs. For a more statistically reliable picture, we took the same distances between NPs in all models: 1, 2 and 3 nm. All parameters and values found are listed in the Table 2, Table 3, Table 4, Table 5, Table 6, Table 7, Table 8, Table 9, Table 10 and Table 11. The dimensions of the surfaces did not affect the obtained values in any way, provided that the number of NPs was sufficient for the generation of LSPR between them (on the whole, dimers were also sufficient). These sizes did not vary significantly, depending on the size of the NPs located on them. Moreover, the height of the surfaces was always the same at 10 nm. In all simulations, it should be implicitly taken into account, that although it is not significant, the SiO_2_ surface influences the SERS performance, due to the influence of the refractive index of the dielectric medium. However, we did not separately analyze the influence of the SiO_2_ surface. Each table is accompanied by an example graph and one example of the *E* field strength simulation. It can be said in advance that the values of *E* and SERS on the substrates were higher than for the single NPs, which is quite justified. Additionally, according to the stars, it associated with a number of difficulties. When arranging the distances between the nearest variable points between the stars, we chose a standard arrangement (as colloidal systems, inside the full surfaces of the beam). The maximum plasmon resonance absorption and it components are simulated and illustrated in Figure 2a,b.

The maximum values for Rh NPs were determined in maximum: extinction: E = 4.6 eV (λ = 270 nm), cross section = 2.2·10^−14^ m^2^; absorption: E = 6.6 eV (λ  = 188 nm), cross section 9.2·10^−15^ m^2^; scattering: E—4.6 eV (λ  = 270 nm), cross section—1.35·10^−15^ m^2^.

The maximum value for Pt NPs were determined in maximum: extinction: E = 4.2 eV (λ = 295 nm), cross section—1.7·10^−14^ m^2^; absorption: E = 4.2 eV (λ = 295 нм), cross section 1·10^−14^ m^2^; scattering: E = 4.4 eV (λ = 281 nm), cross section = 6.9·10^−15^ m^2^.

The graphs of the peaks characterize the optical properties: absorption, correlation with the expected data: the function increases steeply when passing from the visible region ~1.5 eV (~λ = 826 nm) and reaches a maximum in the region close to UV ~4 eV (~λ = 300–310 nm).

The next part of the simulation was carried out for spherical Ag NPs and NPs deposited on a standard SiO_2_ substrate. Hemispheres were used on the substrate for all models of spherical surfaces. The model object was irradiated with two monochromatic laser radiations at wavelengths of 532 nm and 632.8 nm. For the model objects, we varied the radius of the hemispheres (20, 30, 40, and 70 nm). The distance between NPs on the substrates were 1, 2 and 3 nm. Such distances were used for all of the models.

**Figure 3:**
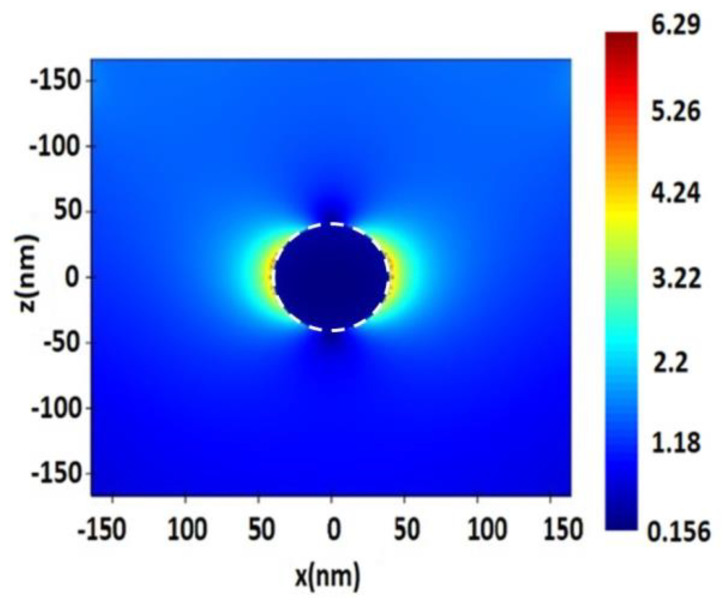
Distribution of electric field value E near the surface of a spherical silver (Ag) NP with a radius of 40 nm, upon excitation by a monochromatic excitation wave λ = 632.8 nm.

**Figure 4:**
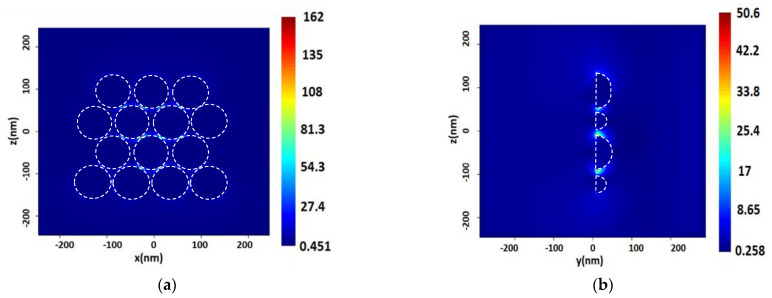
Distribution of the electric field intensity on the surface of spherical silver (Ag) NPs with a radius of 40 nm, a distance between NPs of 2 nm, upon excitation by a monochromatic excitation wave λ = 532 nm in the longitudinal section (plane XZ) (**a**), and in the cross section (plane YZ) (**b**).

In general, for single NPs, there was a correspondence between high electric field values *E* (Table 2, Figure 3 and Figure 4) and high SERS intensities for NPs of a certain geometry. The highest values for *E* and for SERS were obtained on an NP with a radius of 20 nm, irradiated by the laser with a λ = 532 nm wavelength. When using the same laser radiation, no direct dependence was observed: for 30 nm NPs, the values decreased, while for 40 and 70 nm NPs, a slight linear increase occurred. When irradiated with the laser with λ = 632.8 nm wavelength, the NPs showed a linear dependence of the increase in the values of *E* and SERS with an increase in the radius of the spheres.

The surfaces provide *E* and SERS values higher than for single NPs (Table 3). Analyzing the *E* through the distance between NPs on the surface, we revealed the highest value of *E* for NPs with a radius of 70 nm and a distance between hemispheres of 2 nm. In general, the size of NPs on the surfaces affected the value of *E*, increasing with the size of the hemispheres. However, the SERS values in the same model were not high. The highest SERS value was obtained on 30 nm hemispheres with a distance between NPs of 1 nm and for λ = 532 and 632.8 nm monochromatic radiation. Otherwise, no dependence on the sizes or distances between the NPs was observed. This fact can provide correlations with size-dependent characteristics of localized plasmon resonance, which are within the narrow limits from a certain peak. The numerical values of SERS themselves were high enough to provide EF values for such idealized models of the order of 13.

### 3.2. Rh, Pt Single Spheres NPs and Surfaces Consisting of Rh, Pt Hemisphere

**Figure 5:**
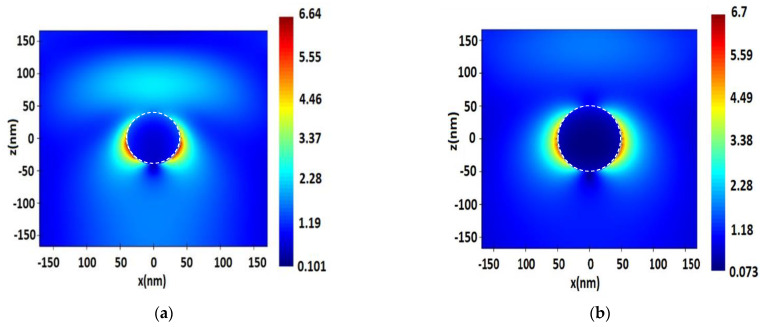
Distribution of electric field value *E* near the surface of a single spherical Pt NP with a radius of 40 nm, upon excitation by a monochromatic wave of λ = 244 nm (**a**) and distribution of electric field strength *E* near the surface of a single spherical Rh NP with a radius of 50 nm, upon excitation by a monochromatic excitation wave of λ = 355 nm (**b**).

The next part addresses the analysis the simulation results for spherical Pt and Rh NPs for the UV plasmonics range. Measurements of 15, 20, 40 and 50 nm for Pt and 35, 40, 50 and 70 nm for Rh are discussed in Table 4 and Table 5 and Figure 5. Model objects were irradiated by laser with λ = 244 and 355 nm wavelengths, correlating with absorption peaks of Pt and Rh in this region. As a result, the differences in the plasmon resonances between these substrates and those with Ag NPs can be compared and evaluated.

The electric field value *E* for Rh and Pt NPs were approximately equal, for Pt, it was slightly higher towards Rh. Furthermore, these values increased linearly with the increasing radius. The SERS values differed significantly for laser radiation at λ = 244 and 355 nm. At λ = 355 nm, the SERS values for Rh and Pt were higher than by an order of magnitude higher, which in general can be associated with the peaks of the plasmon resonant absorption of these metals at a wavelength close to this value. These values for single NPs were even higher than for the Ag ones. In general, SERS values for Rh were higher than for Pt. The highest values for Rh and Pt were obtained on single NPs with a radius of 40 nm, which was the most optimal peak size. Smaller and larger NP sizes gave a linear decrease in the SERS parameters.

**Figure 6:**
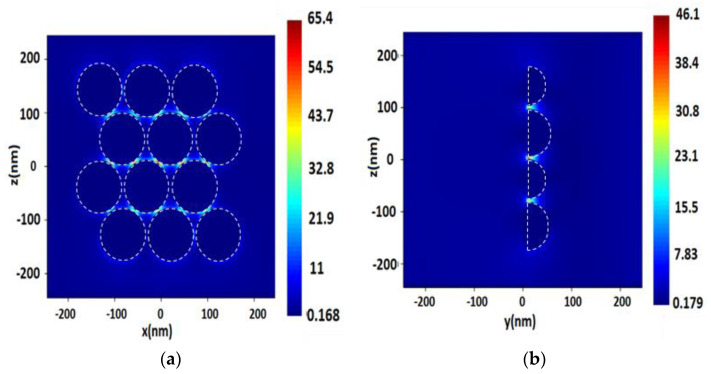
Distribution of electric field value *E* near the surface consisting of a spherical Rh NP with a radius of 50 nm, with a 3 nm distance between NPs, upon excitation by a monochromatic wave of λ = 355 nm in the longitudinal section (plane XZ) (**a**), and in the cross section (plane YZ) (**b**).

**Figure 7:**
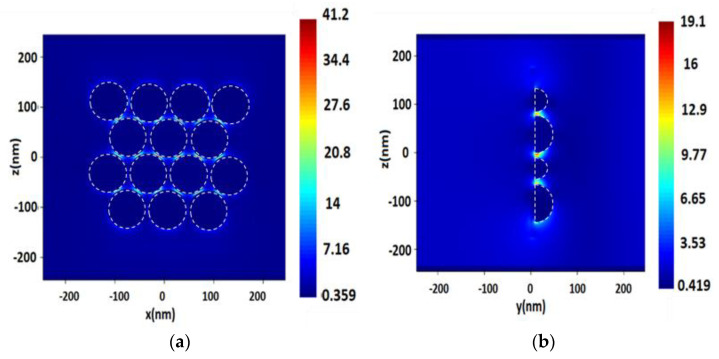
Distribution of the electric field value *E* on the surface consisting of spherical Pt NPs with a radius of 40 nm, with a 2 nm distance between NPs, upon excitation by a monochromatic excitation wave of λ = 244 nm in the longitudinal section (plane XZ) (**a**), and in the cross section (plane YZ) (**b**).

Comparing the values of electric field *E* for Rh and Pt surfaces (Figure 6 and Figure 7, Table 6), we did not observe a significant difference between the results on NPs irradiated by laser with λ = 244 and 355 nm wavelengths. The differences were within units. The distance between NPs near the Rh and Pt surfaces gave a slightly pronounced linear dependence with the increase. When combining the data on Pt and Rh surfaces (Table 6 and Table 7), one could observe a certain dependence of an increase in the electric field strength depending on the size, up to a certain level—50 nm, Rh surfaces with 70 nm hemispheres have already reduced the strength parameter. As with single NPs and surfaces, the differences between the cases of λ = 244 and 355 nm radiation of surfaces for SERS were more than one order of magnitude higher for Rh than for Pt.

Using λ = 244 nm laser excitation, the highest SERS values for Rh and Pt were obtained on surfaces with a hemisphere radius of 50 nm, as in the case of the *E* field value, on average, the intensity values were lower on 70 nm Rh hemispheres. A linear increase in SERS intensity with increasing distance between NPs occurred. For Pt surfaces with 40 and 50 nm hemispheres, the highest SERS values were obtained in this way, in almost all cases. There was a linear increase in SERS with a distance between the spheres. For Pt hemispheres, it is less clear that the SERS values here did not strongly depend on the sizes used, but almost always increased with the distance between the spheres. In comparison with Ag NPs, in terms of electric field strength *E*, Rh and Pt NPs are not far behind—the average values for the strengths for Ag were comparable to high values for Pt and Rh. However, SERS rates are several orders of magnitude higher. The former made us offer conclusions about the degree of difference in the sensitivity of the sensors on these surfaces, especially in the case of detection of single molecules.

### 3.3. Ag single Hexagons NPs and Surfaces Consisting of Ag Hexagons

**Figure 8:**
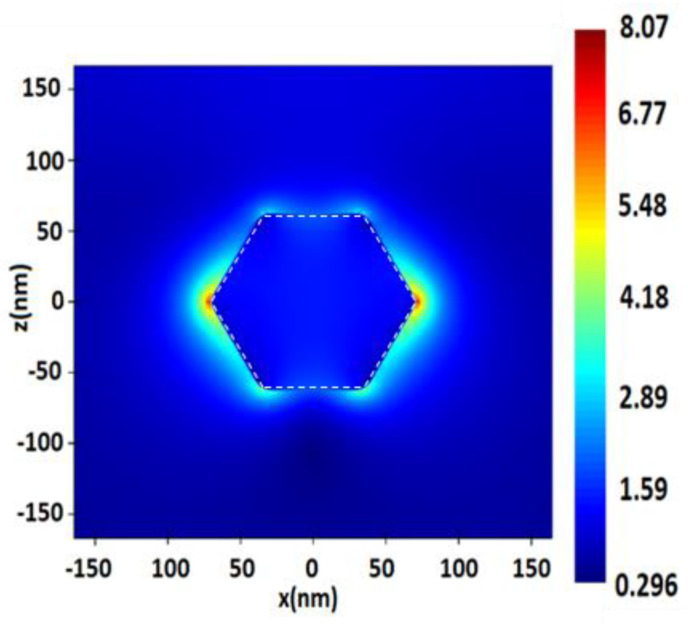
Distribution of electric field value E near the surface of a hexagon silver (Ag) NP with a radius of 70 nm, upon excitation by a monochromatic excitation wave λ = 532 nm.

**Figure 9:**
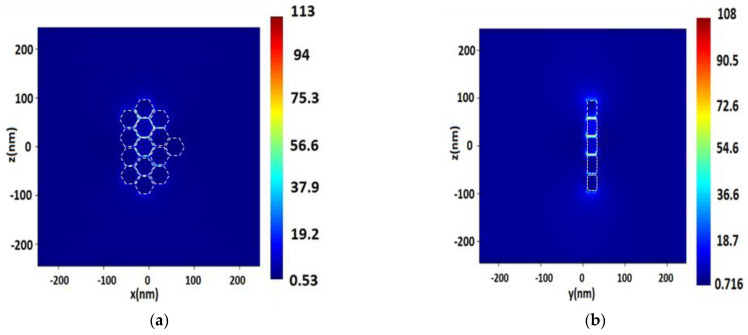
Distribution of the electric field intensity on the surface of hexagonal silver (Ag) NPs with a radius of 40 nm, with a 1 nm distance between NPs of, upon excitation by a monochromatic excitation wave λ = 532 nm in the longitudinal section (plane XZ) (**a**), and in the cross section (plane YZ) (**b**).

Next, we studied hexagons and substrates with hexagonal Ag NPs in the radiation region of 532 and 632.8 nm, for example (Figure 8 and Figure 9). The hexagons were of the following sizes: 20, 40, 50 and 70 nm. The hexagonal surfaces of Ag NPs should first of all be compared with the substrates of spherical Ag NPs.

For single Ag hexagons, the electric field value *E* increased linearly with an increase in the size of NPs. For SERS, the same dependence of the increase in intensity with the size of hexagons was observed (Table 8). The lowest values of electric field *E* were obtained on surfaces with smaller hexagons of 20 nm with a distance of 2 and 3 nm, and for surfaces irradiated at λ = 532 and 632.8 nm. Thus, the highest values were obtained on hexagons of 40 nm in size and with a 2 nm distance between NPs on the surfaces.

### 3.4. Au Single Star NPs and Surfaces Consisting of Ag Stars

**Figure 10:**
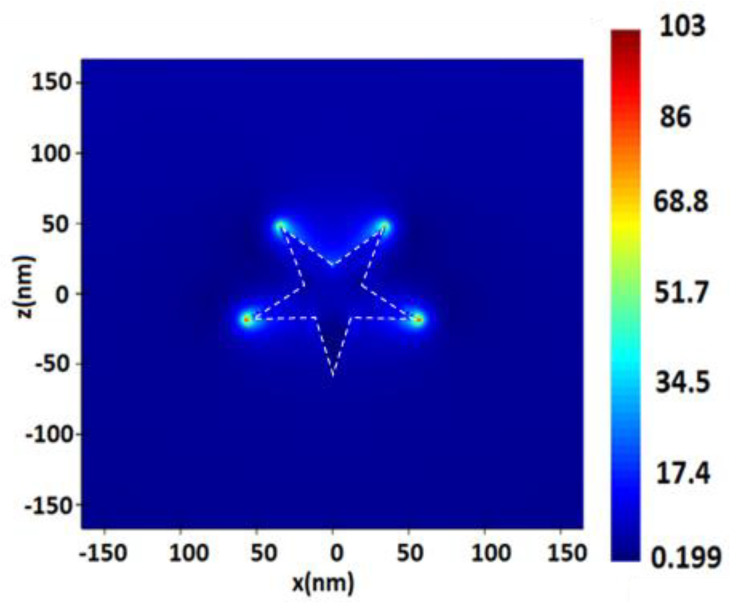
Distribution of electric field value E near the surface of a gold (Au) nanostar with an outer radius of 60 nm, an inner radius of 20 nm, and a height of 10 nm, upon excitation by a monochromatic excitation wave λ = 532 nm.

**Figure 11:**
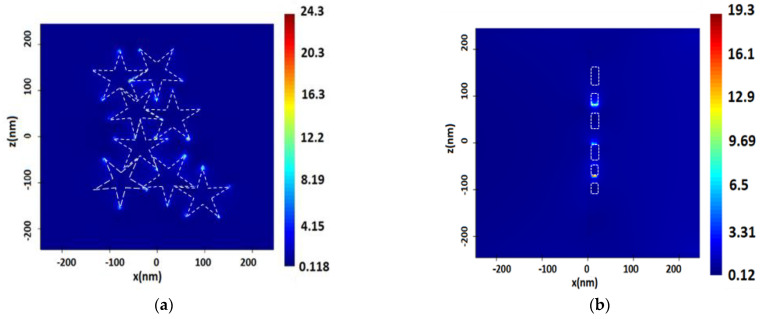
Distribution of the electric field intensity on the surface of gold (Au) nanostars, with an outer radius of 60 nm, an inner radius of 20 nm, a height of 10 nm, with a 1 nm distance between NPs, upon excitation by a monochromatic excitation wave λ = 632.8 nm in the longitudinal section (plane XZ) (**a**), and in the cross section (plane YZ) (**b**).

For Au stars, the localization of LSPR hot spots occurred at the ends of the rays (Figure 8), because of this, it becomes difficult to match the resonant frequency, which is also associated with size characteristics. Unlike other model objects, we took seven dimensional characteristics for the stars, both for single NPs (Table 10, Figure 10) and for surfaces (Table 11, Figure 11). Even with a larger number of model objects, a clear dependence of the values was not observed. On NPs irradiated with monochromatic radiation at 785 nm wavelength, there was a huge spread in the values of field strength *E*. It can only be noted that the largest value of E was obtained for stars with the following dimensional characteristics: a total outer radius of 30 nm, an inner radius of 10 nm and a height of 8 nm at λ = 785 nm laser radiation; a total outer radius of 50 nm, an inner radius of 20 nm, and a height of 8 nm at λ = 632.8 laser radiation.

For the values of SERS at λ = 632.8 nm of laser radiation, a certain, although unclear increase in the dependence on the increase in size of the NPs was obtained. In the near IR range at λ = 785 nm laser radiation, relatively large SERS values were obtained on a stars with large sizes. Comparing with the values of *E*, one can judge about the different contributions to scattering and absorption by NPs (Table 10 and Table 11). On the surfaces, the electric field strengths also gave large spreads in values, as in single stars. On the whole, the values of *E* at λ = 785 nm lasers were, on average, one order higher than those at the λ = 632.8 nm line width. In general, the optimal distance between nanostars, which gave the highest field *E*, on the surface was 2 nm for almost all sizes. The *E* values were relatively average with regards to other surfaces with NPs, and the maximum electric field *E* on the surfaces was lower than the maximum values obtained on some single stars, which can most likely be explained by the overlap of scattered field components. SERS values on surfaces consisting of nanostars also showed significant scatter. Due to the relatively more complex geometry, we cannot observe the interdependence on the sizes or even on the distances between the NPs, all we can say is that the values obtained for 785 nm laser radiation were an order of magnitude higher than for 632.8 nm, and still larger stars gave slightly larger SERS values. As optimal, one can note surfaces with dimensional characteristics of nanostars located on them: the total outer radius is 65 nm, the inner radius is 25 nm, and the height is 10 nm.

## 4. Conclusions

Comparing the SERS values for the presented surfaces with the selected parameters and the lasers we used, the largest values were obtained on spherical Ag surfaces. The SERS values on these surfaces were quite high based on the plasmon absorption peaks of silver in this range. Such values can be used in the detection of resonant excitation of absorbed ligand molecules, in methods requiring high sensitivity at low concentrations. the values of field strength *E* reached maximum for certain geometric parameters with selected roughness coefficient (radius 70 nm, 2 nm distance between hemispheres), which indicates high extinction peaks in these dimensional characteristics.

Hexagonal surfaces produced SERS values slightly lower and only one order of magnitude less EF than the silver ones. The values of field strength *E* for these surfaces, even in some measurements, were higher than for spheres, since the contact surface of local LSPR points were higher than for spherical ones, which could affect the generally high extinction parameters.

In this paper we calculated the SERS values for UV and visible range plasmon-active surfaces and single NPs. It has been shown that Rh surfaces can be compared with gold nanostars obtained values approaching the UV range and visible range respectively. NPs differs with only one order of SERS theoretical magnitude. The Pt surfaces in the UV range provided the lowest values, but not the smallest when considering the conditions of use of these surfaces, as, for example, the values obtained in metal-enhanced fluorescence spectroscopy methods. Surfaces with stars require more careful study and more statistical data should be analyzed both in terms of geometric parameters and in the study of optical characteristics. The possibilities for a theoretical approach for single NP and planar surfaces modeling to evaluate optimal field amplification and light scattering parameters have been shown. The presented approach could be applied as a basis for performing methods of controlled synthesis for colloidal and planar metal-based biocompatible optical sensors for UV and deep-UV plasmonics.

## Figures and Tables

**Table 1:** Dielectric constant parameters for NPs.

Excitation Wavelength λ, nm	Structure Type	Re (ε)	Im (ε)
244	Rh NPs	−4.64	3.62
	Pt NPs	−1.12	4.69
355	Rh NPs	−12.75	7.78
	Pt NPs	−3.97	8.18
532	Ag NPs	−12.14	1.74
632.8	Ag NPs	−18.66	2.33
	Gold NS	−10.8	0.795
785	Gold NS	−21.64	0.74

**Table 2:** Electric field parameters for single Ag NPs.

Sphere Ag Radius	Excitation Wavelength, nm					
	532	632.8	532	632.8	532	632.8
	Local Maximum of Electric Field *E, V/m*		SERS Signal Intensity, a.u.		Enhancement Coefficient |*E*/*E*_0_|^4^	
20	14.6	4.52	1600	658	2.57·10^6^	4.33·10^5^
30	2.76	5.25	678	860	4.59·10^5^	7.39·10^5^
40	3.43	6.29	984	1300	9.69·10^5^	1.68·10^6^
70	4.66	7.71	1030	1370	1.07·10^6^	1.88·10^6^

**Table 3:** Electric field parameters for the surfaces consisting of Ag hemisphere.

Hemisphere Ag Radius	L	Excitation Wavelength, λ—nm					
		532	632.8	532	632.8	532	632.8
		Local Maximum of Electric Field *E, V/m*		SERS Signal Intensity, a.u.		Enhancement Coefficient |*E*/*E*_0_|^4^	
20	1	34.5	19.2	4.88·10^6^	8.54·10^5^	2.38·10^13^	7.29·10^11^
	2	33	18.1	6.61·10^5^	5.95·10^5^	4.36·10^11^	3.54·10^11^
	3	31.4	17.4	3.92·10^5^	4.2·10^5^	1.54·10^11^	1.76·10^11^
30	1	58	32.1	7.6·10^6^	1.21·10^6^	5.78·10^13^	1.45·10^12^
	2	69.9	38.1	1.59·10^6^	3.03·10^5^	2.54·10^12^	9.16·10^10^
	3	68	37.3	2.44·10^6^	4.28·10^5^	5.95·10^12^	1.83·10^11^
40	1	121	63.9	1.62·10^6^	2.72·10^5^	2.62·10^12^	7.37·10^10^
	2	162	83.7	2.04·10^6^	3.74·10^5^	4.15·10^12^	1.4·10^11^
	3	112	59.3	2.55·10^6^	7.03·10^5^	6.48·10^12^	4.94·10^11^
70	1	122	59.3	7.81·10^5^	3.38·10^5^	6.1·10^11^	1.15·10^11^
	2	240	109	3.32·10^5^	3.25·10^5^	1.1·10^11^	1.05·10^11^
	3	149	68.5	3.51·10^5^	4.31·10^5^	1.23·10^11^	1.86·10^11^

**Table 4:** Electric field parameters for single Pt NPs.

Hemisphere Pt Radius	Excitation Wavelength, λ, nm					
	244	355	244	355	244	355
	Local Maximum of Electric Field *E, V/m*		SERS Signal Intensity, a,u		Enhancement Coefficient |*E*/*E*_0_|^4^	
15	4.96	4.97	114	4270	12.900	1.83·10^7^
20	5.25	5.26	137	4880	18.900	2.38·10^7^
40	6.64	6.66	221	8120	49.000	6.6·10^7^
50	6.69	6.7	167	7120	28.000	5.06·10^7^

**Table 5:** Electric field parameters for single Rh and Pt NPs.

Sphere Rh Radius	Excitation Wavelength, λ—nm					
	244	355	244	355	244	355
	Local Maximum of Electric Field *E, V/m*		SERS Signal Intensity, a,u		Enhancement Coefficient |*E*/*E*_0_|^4^	
35	5.99	6.01	262	7570	68.800	5.74·10^7^
40	6.64	6.66	341	9500	116.000	9.02·10^7^
50	6.67	6.7	245	8500	60.200	7.22·10^7^
70	7.36	7.38	124	4830	15.500	2.33·10^7^

**Table 6:** Electric field parameters for the surfaces consisting of Pt NPs.

Hemisphere Pt Radius	L	Excitation Wavelength, λ—nm					
		244	355	244	355	244	355
		Local Maximum of Electric Field *E, V/m*		SERS Signal Intensity, a.u.		Enhancement Coefficient |*E*/*E*_0_|^4^	
15	1	14.1	14.1	223	2040	4.98·10^4^	4.18·10^6^
	2	16.1	16.1	293	2580	8.6·10^4^	6.67·10^6^
	3	16.7	16.7	327	2940	1.07·10^5^	8.66·10^6^
20	1	19.7	19.8	399	1950	1.59·10^5^	3.81·10^6^
	2	22.9	22.9	565	2740	3.2·10^5^	7.5·10^6^
	3	23.7	23.7	616	3050	3.79·10^5^	9.28·10^6^
40	1	37	37.3	1370	3850	1.48·10^7^	4.87·10^5^
	2	41.2	41.4	1960	4820	2.87·10^6^	2.32·10^7^
	3	44.2	44.6	1960	2940	3.83·10^6^	8.66·10^6^
50	1	37.5	37.7	1410	3720	1.98·10^6^	1.39·10^7^
	2	48.1	48.5	2130	4030	5.35·10^6^	1.62·10^7^
	3	53.1	53.4	2820	4810	7.43·10^6^	2.31·10^7^

**Table 7:** Electric field parameters for the surfaces consisting of Rh NPs.

Hemisphere Rh Radius	L	Excitation Wavelength, λ—nm					
		244	355	244	355	244	355
		Local Maximum of Electric Field *E, V/m*		SERS Signal Intensity, a,u.		Enhancement Coefficient |*E*/*E*_0_|^4^	
35	1	16.6	16.1	787	3440	6.19·10^5^	1.18·10^7^
	2	22	20.9	1140	5960	1.3·10^6^	3.55·10^7^
	3	21.4	20.4	1290	7140	1.66·10^6^	5.09·10^7^
40	1	44.9	41	2480	14000	6.13·10^6^	1.97·10^8^
	2	52.2	48.9	3080	19600	9.46·10^6^	3.84·10^8^
	3	54.3	51.5	3800	23100	1.44·10^7^	5.32·10^8^
50	1	37.5	35	2440	13500	5.95·10^6^	1.82·10^8^
	2	63.4	59.4	4020	15600	1.62·10^7^	2.42·10^8^
	3	69.9	65.4	4880	20400	2.39·10^7^	4.16·10^8^
70	1	54	50.1	2910	17800	8.48·10^6^	3.18·10^8^
	2	56.9	53.6	3690	16300	1.36·10^7^	2.65·10^8^
	3	61	57.7	3900	22300	1.52·10^7^	4.97·10^8^

**Table 8:** Electric field parameters for hexagonal Ag NPs.

Hexagon Ag Radius	Excitation Wavelength, λ—nm					
	532	632.8	532	632.8	532	632.8
	Local Maximum of Electric Field *E, V/m*		SERS Signal Intensity, a.u		Enhancement Coefficient |*E*/*E*_0_|^4^	
20	2.18	4.4	525	645	2.75·10^5^	4.16·10^5^
40	3.86	7.24	1440	2010	2.07·10^6^	4.04·10^6^
50	4.89	9.13	2650	3710	7.01·10^6^	1.38·10^7^
70	8.07	14.3	6890	8840	4.74·10^7^	7.82·10^7^

**Table 9:** Electric field parameters for hexagonal Ag surfaces.

Hexagon Ag Radius	L	Excitation Wavelength, λ, nm					
		532	632.8	532	632.8	532	632.8
		Local Maximum of Electric Field *E, V/m*		SERS Signal Intensity, a,u.		Enhancement Coefficient |*E*/*E*_0_|^4^	
20	1	158	88	8.92·10^5^	3.9·10^5^	7.96·10^11^	1.52·10^11^
	2	28.3	15.8	5.8·10^5^	3.77·10^5^	3.37·10^11^	1.42·10^11^
	3	27.2	15.4	8.15·10^5^	3.12·10^5^	6.64·10^11^	9.75·10^10^
40	1	113	62.7	6.85·10^5^	2.17·10^5^	4.69·10^11^	4.69·10^10^
	2	210	132	4.08·10^5^	9.09·10^4^	1.66·10^11^	8.26·10^9^
	3	73	32.1	6.21·10^5^	1.39·10^5^	3.85·10^11^	1.92·10^10^
50	1	47.4	24.3	3.68·10^5^	1.38·10^5^	1.36·10^11^	1.9·10^10^
	2	166	86.3	2.19·10^5^	1.45·10^5^	4.81·10^10^	2.1·10^10^
	3	94.4	50.5	3.95·10^5^	1.52·10^5^	1.56·10^11^	2.3·10^10^
70	1	76.6	39.6	1.76·10^5^	1.45·10^5^	3.11·10^10^	2.09·10^10^
	2	160	86.6	2.18·10^5^	7.96·10^4^	4.75·10^10^	6.33·10^9^
	3	181	95.2	1.5·10^5^	1.18·10^5^	2.25·10^10^	1.4·10^10^

**Table 10:** Electric field parameters calculated for Au nanostars.

Stars Au	Excitation Wavelength, λ, nm					
	632.8	785	632.8	785	632.8	785
	Local Maximum of Electric Field *E, V/m*		SERS Signal Intensity, a,u.		Enhancement Coefficient |*E*/*E*_0_|^4^	
Outer radius 30 nm, Inner radius 10 mn, Height 8 nm	11.9	116	1060	13500	1.13·10^6^	1.82·10^8^
Outer radius 40 nm, Inner radius 10 mn, Height 8 nm	8.76	17.2	982	10900	9.64·10^5^	1.18·10^8^
Outer radius 45 nm, Inner radius 15 mn, Height 8 nm	22.1	25.5	1390	17600	1.92·10^6^	3.09·10^8^
Outer radius 50 nm, Inner radius 20 mn, Height 10 nm	39.9	49.1	9900	2510	4.16·10^6^	9.8·10^7^
Outer radius 60 nm, Inner radius 20 mn, Height 10 nm	17.2	103	1640	10600	2.68·10^6^	1.13·10^8^
Outer radius 65 nm, Inner radius 25 nm, Height 10 nm	24.8	156	1870	77600	3.48·10^6^	6.03·10^9^
Outer radius 75 nm, Inner radius 25 nm, Height 10 nm	23.4	40.1	3140	4.95·10^5^	9.84·10^6^	2.45·10^11^

**Table 11:** Electric field parameters calculated for Au surfaces consisting of nanostars.

Stars Au	L	Excitation Wavelength, λ—nm					
		632.8	785	632.8	785	632.8	785
		Local Maximum of Electric Field *E, V/m*		SERS Signal Intensity, a,u.		Enhancement Coefficient |*E*/*E*_0_|^4^	
Outer radius 30 nm, Inner radius 10 mn, Height 8 nm	1	52.5	79.3	7540	19700	5.68·10^7^	3.88·10^8^
	2	37.9	63.4	5440	11900	2.96·10^7^	1.42·10^8^
	3	48	70.4	5900	15700	3.48·10^7^	2.48·10^8^
Outer radius 40 nm, Inner radius 10 mn, Height 8 nm	1	16.8	20.9	2530	18800	6.4·10^6^	3.55·10^8^
	2	27	36.6	2450	18400	6.01·10^6^	3.38·10^8^
	3	17.5	23.7	1650	5490	2.73·10^6^	3.02·10^7^
Outer radius 45 nm, Inner radius 15 mn, Height 8 mn	1	42.4	62.8	5570	19600	3.13·10^7^	3.83·10^8^
	2	57.2	87.3	7590	23800	5.76·10^7^	5.67·10^8^
	3	22.4	32.5	4770	36900	2.27·10^7^	1.36·10^9^
Outer radius 50 nm, Inner radius 20 mn, Height 10 nm	1	49.1	69	15,600	35400	2.45·10^8^	1.25·10^9^
	2	53.8	95.1	13,400	28800	1.79·10^8^	8.29·10^8^
	3	37.9	53.8	18,000	27700	3.23·10^8^	7.7·10^8^
Outer radius 60 nm, Inner radius 20 mn, Height 10 nm	1	24.3	35	11,700	51300	1.36·10^8^	2.64·10^9^
	2	42.4	62.9	7880	56100	6.21·10^7^	3.15·10^9^
	3	29.4	39	7180	41100	5.16·10^7^	1.69·10^9^
Outer radius 65 nm, Inner radius 25 mn, Hei ght 10 nm	1	61.3	87.3	13,900	1.52·10^5^	1.92·10^8^	2.32·10^10^
	2	79.8	117	7130	45800	5.08·10^7^	2.09·10^9^
	3	31.1	46.5	9690	43800	9.38·10^7^	1.92·10^9^
Outer radius 75 nm, Inner radius 25 mn, Hei ght 10 nm	1	30.3	44.9	11,400	11000	1.29·10^8^	1.36·10^10^
	2	34.7	42.2	9180	46710	8.42·10^7^	2.18·10^9^
	3	27.9	36.4	11,500	23300	1.33·10^8^	5.42·10^8^

## Data Availability

Data is contained within the article.

## Appendix A

clear;
closeall;
*script conversion to the second degree of the maximum values of the electric field strength*
#get the electric intensity
exz=pinch(getelectric(“XZ”));
eyz=pinch(getelectric(“YZ”));
#find the maximum
mxz=max((exz)^2);
myz=max((eyz)^2);
# get some raw *(data numerical values obtained as a matrix on the axes)*
x1=getdata(“XZ”,”x”);
z1=getdata(“XZ”,”z”);
y2=getdata(“YZ”,”y”);
z2=getdata(“YZ”,”z”);
f=getdata(“YZ”,”f”);
*# [finding the gain (EF and intensity) through the calculation of the maximum values in the matrix]*
EFxz=exz^2;#enhancement factor
index = find(EFxz,mxz);
# convert index to row, col *indices (finding numerical values by accessing indices at grid nodes)*
matrix_size = size(EFxz);
indices = matrix(length(matrix_size));
# do for each dimension
for (i = 1:length(matrix_size)) {
mod_dividend = index;
mod_divisor = matrix_size(i);
mod_remainder = mod(mod_dividend,mod_divisor);
if (mod_remainder == 0) { mod_remainder = matrix_size(i); }
indices(i) = mod_remainder;
# remove this dimension from further calculations
index = (index+(matrix_size(i)-mod_remainder))/matrix_size(i);
}
?”max at x1 =“+num2str(x1(indices(1))*1e6)+” um”;
?”multi indice access: EF factor xz (“+num2str(indices(1))+”,”+
num2str(indices(2))+”,”+
num2str(indices(3))+”)=“+
num2str(EFxz(indices(1),indices(2),indices(3)));
# users may need to modify those values in order to have proper view of the resulting images *(image size)*
zmin=-200e-9;
zmax= 200e-9;
xym = 150e-9;
#
nx1=find(x1,-xym)-1;
nx2=find(x1, xym)+1;
nz1=find(z1,zmin)-1;
nz2=find(z1,zmax)+1;
image(x1(nx1:nx2)*1e9,z1(nz1:nz2)*1e9,EFxz(nx1:nx2,nz1:nz2,indices(3)),”x nm”,”z nm”,”EF xz”);
image(x1(nx1:nx2)*1e9,z1(nz1:nz2)*1e9,exz(nx1:nx2,nz1:nz2,indices(3)),”z nm”,”z nm”,”Intensity xz”);
# in another cross section *(the same actions for the plane YZ)*
EFyz=eyz^2;
index = find(EFyz,myz);
# convert index to row, col indices
matrix_size = size(EFyz);
indices = matrix(length(matrix_size));
# do for each dimension
for (i = 1:length(matrix_size)) {
mod_dividend = index;
mod_divisor = matrix_size(i);
mod_remainder = mod(mod_dividend,mod_divisor);
if (mod_remainder == 0) { mod_remainder = matrix_size(i); }
indices(i) = mod_remainder;
# remove this dimension from further calculations
index = (index+(matrix_size(i)-mod_remainder))/matrix_size(i);
}
?”multi indice access: EF factor yz (“+num2str(indices(1))+”,”+
num2str(indices(2))+”,”+
num2str(indices(3))+”)=“+
num2str(EFyz(indices(1),indices(2),indices(3)));
?”max at y1 =“+num2str(y2(indices(1))*1e6)+” um”;
ny1=find(y2,-xym)-1;
ny2=find(y2, xym)+1;
nz1=find(z2,zmin)-1;
nz2=find(z2,zmax)+1;
image(y2(ny1:ny2)*1e9,z2(nz1:nz2)*1e9,EFyz(ny1:ny2,nz1:nz2,indices(3)), “y nm”,”z nm”,”EF yz”);
image(y2(ny1:ny2)*1e9,z2(nz1:nz2)*1e9,eyz(ny1:ny2,nz1:nz2,indices(3)),”y nm”,”z nm”,”Intensity yz”);
